# Texture-enhanced thermography for joint inflammation detection using consumer-grade thermal cameras

**DOI:** 10.1371/journal.pone.0354709

**Published:** 2026-07-29

**Authors:** Shaye Kivity, Dima Bykhovsky, Nadav Sheffer, Keren Netzer, Yael Pri-Paz Basson, Oshrat Tayer-Shifman, Rotem Sivan, Netzah Calamaro, Rami Ginaime, Eli Flaxer, Sara Naftali, Anat Ratnovsky, Lior Luria, Oshrit Hoffer

**Affiliations:** 1 Rheumatology Unit, Meir Medical Center, Kfar Saba, Israel; 2 Sackler Faculty of Medicine, Tel-Aviv University, Tel Aviv, Israel; 3 Electrical and Electronics Engineering, Shamoon College of Engineering, Beer-Sheva, Israel; 4 School of Medical Engineering, Afeka Academic College of Engineering in Tel Aviv, Tel Aviv, Israel; 5 School of Electrical Engineering, Afeka Academic College of Engineering in Tel Aviv, Tel Aviv, Israel; Transilvania University of Brasov: Universitatea Transilvania din Brasov, ROMANIA

## Abstract

**Background:**

Early identification of active synovitis is essential for treat-to-target management in inflammatory arthritis. While MRI and ultrasound are highly sensitive, their clinical use is constrained by high costs, operator dependence, and lack of portability. Infrared thermography (IRT) is rapid and contactless, but conventional temperature-based approaches are sensitive to ambient conditions and inter-individual baseline variability, and contralateral comparison is unreliable in bilaterally involved disease. This study evaluates whether consumer-grade thermal cameras, augmented with texture-based features and complementary differencing strategies, can detect and differentiate joint inflammation.

**Methods:**

Thermal images of knees, ankles, wrists, and metacarpophalangeal (MCP) joints were acquired from 239 participants (167 with inflammatory arthritis, osteoarthritis (OA), or fibromyalgia; 72 healthy controls). Beyond absolute temperature, higher-order texture features (e.g., entropy, skewness) were extracted to quantify spatial heterogeneity in heat distributions. To reduce environmental and inter-individual variability, two normalization strategies were applied: contralateral left–right (L-R) differencing for unilateral disease and a novel anterior–posterior (A-P) differencing for bilateral or subclinical presentations. Group comparisons used the Mann-Whitney U test (*p* < 0.05), and effect sizes were summarized using Cohen’s *d*.

**Results:**

Compared with non-differenced features, contralateral differencing markedly strengthened discrimination for unilateral inflammation, with very large effects at the knee (*d* = 2.15) and ankle (*d* = 1.83). Texture features, specifically entropy and skewness, outperformed absolute temperature in detecting latent (subclinical) wrist inflammation via A-P differencing (d=−1.11). Thermographic signatures also supported differential characterization, distinguishing inflammatory arthritis from OA (d=−1.49) and fibromyalgia (d=−1.17). Furthermore, A-P gradients showed anatomical specificity, with inflammation reducing gradients in wrists but increasing them in MCP joints (*d* = 0.64).

**Conclusion:**

Texture-enhanced thermography, combined with L-R and A-P differencing, shows promise as an accessible approach for identifying active and subclinical joint inflammation. This approach addresses the limitations of bilateral disease in traditional thermography and widens the clinical applicability of IRT screening.

## Introduction

Arthritis comprises a heterogeneous group of inflammatory and degenerative disorders that can cause pain, functional limitation, and progressive joint damage [1]. In inflammatory arthritis, management is commonly guided by a treat-to-target approach, where early identification of active synovitis and timely escalation of therapy are critical for the prevention of structural progression and disability [2]. A recurring clinical challenge is that physical examination can be insensitive to low-grade or early disease and does not reliably distinguish localized joint inflammation from broader systemic or non-inflammatory pain phenotypes.

Ultrasound and magnetic resonance imaging (MRI) improve sensitivity for detecting synovitis, tenosynovitis, and periarticular inflammation [3–5]. However, the usefulness of these techniques is limited by cost, access, operator dependence, and lengthy acquisition or interpretation times. MRI is costly and time-intensive, limiting feasibility for repeated monitoring, while ultrasound is operator-dependent and can be time-consuming when multiple sites must be scanned, with additional constraints in settings with limited imaging access [6]. These limitations motivate interest in accessible, non-invasive methods that can complement clinical evaluation in routine practice.

Infrared thermography (IRT) offers a rapid, contactless solution that quantifies skin-surface temperature and can detect arthritis by identifying inflammation-associated temperature changes. Inflammatory mediators increase local blood flow, producing focal warming that thermal imaging resolves at the skin surface [7–14]. A recent systematic review of IRT in rheumatic diseases positions it as an emerging complementary tool with high portability and low per-scan cost [15], and an earlier systematic synthesis across inflammatory and degenerative joint diseases concluded that thermography reliably separates active synovitis from non-inflamed joints, though with marked heterogeneity in acquisition protocols across studies [12]. For knee osteoarthritis, a scoping review of thermal thresholds identifies inter-limb temperature differences of roughly $0.5^{\circ }$C as a commonly reported discriminative cut-off [13].

Joint-focused studies in rheumatoid arthritis (RA) have quantified the relationship between thermographic parameters and ultrasound-detected inflammation across several anatomical sites (elbow, wrist, MCP, knee), and complementary work has advanced composite IRT-based disease-activity indices and demonstrated feasibility on consumer-grade hardware in home settings. Table 1 summarizes the headline correlation, AUROC, and sensitivity/specificity figures reported across these studies [6,12–19]. Collectively, these results establish that IRT carries a quantitative signal for arthritis-related inflammation, while also motivating the methodological extensions pursued in the present study, namely texture-based descriptors and dual differencing strategies that are robust to the variability of consumer-grade acquisition.

**Table 1:** Reported quantitative performance of infrared thermography (IRT) in arthritis-related studies. Summary of correlation coefficients (*r*), AUROC values, and sensitivity/specificity figures from recent joint-focused studies and systematic/scoping reviews. US = ultrasound; PD = power Doppler.

Study	Joint / Site	Reference standard	Key quantitative finding
[6]	Wrist	US greyscale / PD	*r* = 0.33 to 0.37 (greyscale); *r* = 0.43 to 0.48 (PD)
[12]	Multiple	Systematic review	IRT separates active synovitis from non-inflamed joints; heterogeneous protocols across included studies
[13]	Knee (OA)	Scoping review	Inter-limb $\Delta T\approx 0.5^{\circ }$C used as discriminative threshold
[14]	MCP	US-defined subclinical synovitis	Significant thermal/US association at MCP joints
[15]	Multiple	Systematic review	Positions IRT as a portable, low-cost complementary tool for rheumatic disease assessment
[16]	Elbow	US greyscale / PD	*r* = 0.39 to 0.42 (greyscale); *r* = 0.40 to 0.55 (PD)
[17]	Knee	US-detected inflammation	AUROC 0.63 to 0.82; side-to-side $T_{\max}$ difference most discriminative
[18]	Hands (RA)	CDAI / SDAI; expert composite	ThermoDAI vs. CDAI *r* = 0.81; vs. SDAI *r* = 0.83; ML index external AUROC ≈0.67
[19]	Multiple (paediatric, home)	Clinical exam	Specificity 79% to 85% with temperature-based decision rules

Despite these advances, several gaps that limit translation into routine screening or monitoring remain. First, raw temperature measurements are sensitive to ambient conditions and to inter-individual baseline variation (e.g., age, microvascular tone, systemic inflammation); this can obscure joint-specific disease signals. Second, many studies evaluate a single anatomical site or narrowly defined cohorts, leaving uncertainty about joint dependence and generalizability. Third, most pipelines emphasize summary temperature statistics, whereas the spatial distribution of heat may encode additional information about inflammatory heterogeneity. Fourth, contralateral comparison, while effective for unilateral disease, cannot be applied when both joints are affected, which is a common presentation in rheumatoid arthritis and other symmetric inflammatory conditions. Finally, although machine-learning thermographic indices have shown promise [18], there remains a practical need for feature engineering strategies that are robust to deployment with consumer-grade cameras and that generalize across joints and disease states.

To address these gaps, the present study evaluates whether consumer-grade thermography can reliably discriminate clinically inflamed from non-inflamed joints. We propose a methodology that augments compact thermal imaging with three key analytical strategies: (i) the extraction of texture-based descriptors to capture spatial heat heterogeneity beyond summary statistics; (ii) contralateral (L-R) differencing to mitigate inter-individual and environmental confounding in unilateral disease; and (iii) a novel anterior-posterior (A-P) differencing approach to detect inflammation in bilateral or subclinical presentations where contralateral comparison is not viable. By evaluating multiple joint sites (knees, ankles, wrists, and metacarpophalangeal joints) across diverse clinical states (inflammation, remission, osteoarthritis, and fibromyalgia), we aim to evaluate a robust, cost-effective framework that tolerates real-world acquisition variability and could complement point-of-care arthritis assessment.

## Materials and methods

### Study cohort

The study was approved by the Institutional Review Board (IRB) of Meir Medical Center (Approval No. MMC-22–108). All procedures were performed in accordance with the relevant guidelines and regulations. Informed consent was obtained from all participants prior to participation in the study. Participants were recruited from the Rheumatology Clinic at Meir Medical Center, Israel, between October 2022 and August 2023.

The cohort included individuals diagnosed with inflammatory arthritis (rheumatoid arthritis, psoriatic arthritis, undifferentiated arthritis), OA, or fibromyalgia, along with healthy volunteers. Patients with inflammatory arthritis were classified into inflammation (clinical evidence of active synovitis at the imaged joint) or remission (no clinical synovitis). Clinical classification was determined by two expert rheumatologists, whose assessments were in agreement. The final dataset comprised 239 participants: 167 patients and 72 healthy controls. Demographic characteristics are summarized in Table 2.

**Table 2:** Demographic characteristics of the study cohort.

Characteristic	Total	Healthy Controls	Patients
*N*	239	72 (30.1%)	167 (69.9%)
Age (years)			
Mean ± SD	55.7 ± 17.6	46.1 ± 18.0	59.9 ± 15.8
Median	57.0	43.5	63.0
Range	19–94	19–84	20–94
*Sex*			
Female	163 (68.2%)	46 (63.9%)	117 (70.1%)
Male	76 (31.8%)	26 (36.1%)	50 (29.9%)

### Thermal imaging acquisition protocol

The goal of the protocol was to minimize environmental noise and improve reproducibility. Thermal images were acquired using either a FLIR C5 compact thermal camera or a FLIR ONE Pro smartphone attachment. For each participant, the hands, ankles, and knees were imaged bilaterally. Room temperature was maintained at 21–25 °C and humidity at 37–55%, and subject metadata (e.g., age and temperature) were recorded. Participants removed clothing and jewelry covering the imaged joints. The camera was held perpendicular to the joint at a uniform distance of about 50 cm to standardize geometry across acquisitions. Participants rested for 15 minutes in a temperature-controlled waiting room before imaging and were asked to refrain from physical activity, smoking, eating, and drinking during this period.

To ensure a comprehensive thermal map, both anterior (palmar) and posterior (dorsal) views were captured for knees and hands. For ankle joints and the specific OA subgroup, the protocol focused exclusively on anterior views. Some participants were unable to complete certain imaging orientations.

### MRI evaluation

To validate thermal imaging findings against a gold standard, MRI evaluation was performed on patients with suspected inflammatory arthritis in wrists and inconclusive clinical assessment. The MRI scans focused on detecting early (subclinical) inflammatory changes, such as synovitis, bone marrow edema, and tenosynovitis, which are known indicators of active arthritis even at subclinical stages. MRI evaluations (see Table 2 in S1 File) with 17 wrist and 11 metacarpophalangeal (MCP) patients were used for paired thermal-MRI comparisons.

### Region-of-Interest (ROI) segmentation and feature extraction

Thermal images were analyzed in Matlab. Skin emissivity was set to ϵ=0.98 for temperature conversion. Regions-of-interest (ROIs) encompassing the joint area were delineated, and mean, maximum, and minimum ROI temperatures were manually extracted. In addition, texture descriptors were computed from the ROI thermal matrix, including entropy, local entropy, skewness, kurtosis, temperature variance, contrast, correlation, homogeneity, and energy.

### Differencing analysis

#### Contralateral differencing.

**Fig 1:**
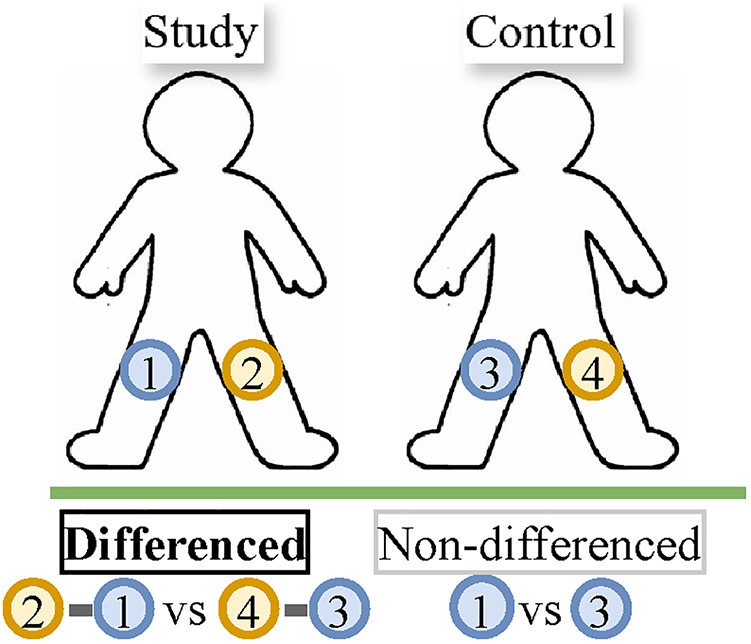
Differencing strategy. Thermal features from corresponding joints (e.g., left versus right knee) were compared within individuals in both study and control groups. The resulting contralateral intra-subject differences helped isolate inflammation-driven asymmetries.

To account for individual differences and environmental fluctuations, a contralateral differencing strategy was employed: each feature extracted from the affected (or suspected) joint was subtracted from the corresponding feature of the contralateral joint in the same individual (Fig 1),


$$\begin{array}{c} \Delta f_{L-R}=|f_{L}-f_{R}|, \end{array}$$ (1)

with $\Delta f_{L-R}$ used for further statistical analysis. This approach yields a normalized measure of asymmetry and reduces the influence of systemic factors such as ambient temperature or metabolic state.

#### Anterior-posterior (A-P) differencing.

The study used A-P difference patterns between joint sites,


$$\begin{array}{c} \Delta f_{A-P}=f_{A}-f_{P}. \end{array}$$ (2)

This was applied in a similar manner to the contralateral differencing.

### Statistical analysis

All extracted features were compiled into a structured dataset. Descriptive statistics were calculated, and normality was assessed via the Shapiro-Wilk test. Because most distributions deviated from normality, the Mann-Whitney U test was used to compare inflamed versus control joints. Statistical significance was set at *p* < 0.05.

To quantify the magnitude of the differences between groups, Cohen’s *d* effect sizes were calculated. Effect sizes were categorized as small (0.2≤|d|<0.5), medium (0.5≤|d|<0.8), large (0.8≤|d|<1.2), and very large (1.2≤|d|).

The analysis was performed using Python with the scipy package.

### Workflow

**Fig 2:**
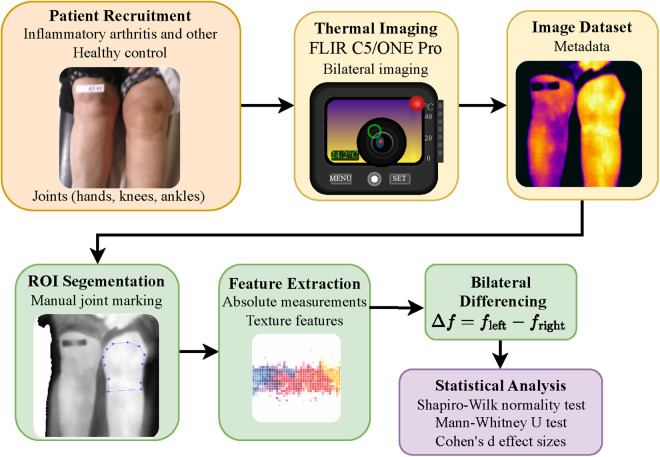
The workflow of the study. This figure illustrates the sequential stages of the experimental process, from participant recruitment through statistical analysis.

The experimental workflow (Fig 2) proceeded as follows: participants were recruited and categorized into clinical groups; bilateral thermal images were captured under controlled environmental conditions; joint-specific regions of interest were manually delineated to isolate the target anatomy; first-order temperature metrics and higher-order texture descriptors were extracted and subjected to contralateral and anterior-posterior differencing; finally, group-wise comparisons and effect-size calculations identified discriminative features.

**Fig 3:**
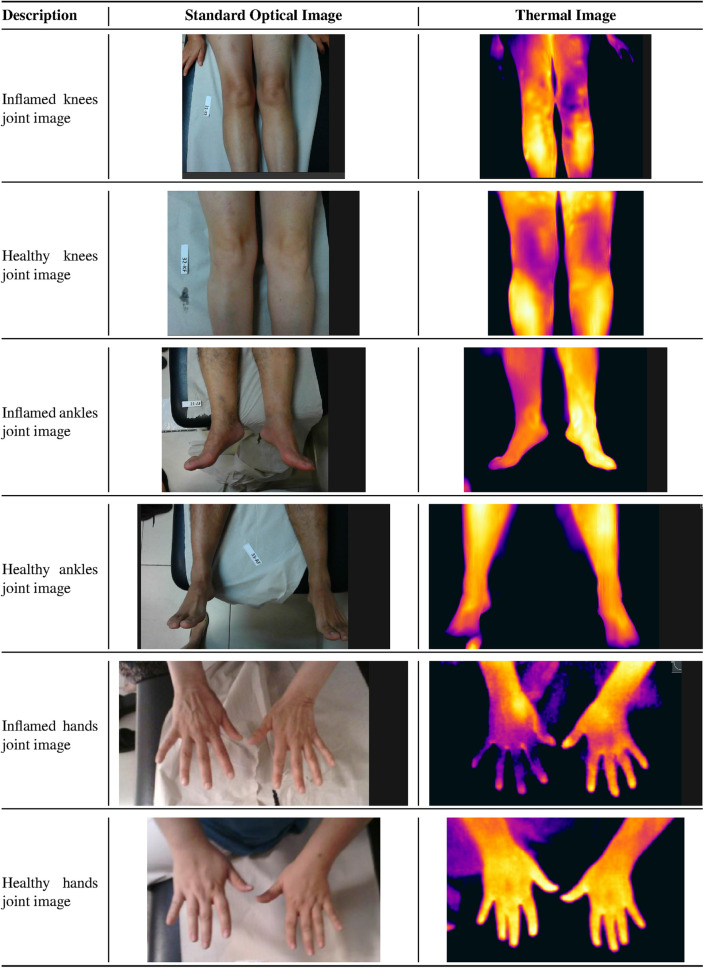
Representative optical and thermal images. Optical (left) and thermal (right) views of knees, ankles, and hands. Joints with confirmed arthritis exhibit significant localized heat and noticeable temperature differences between sides. In contrast, joints from healthy controls display a more consistent and even temperature distribution.

Fig 3 illustrates representative optical and thermal images of knees, ankles, and wrists. Joints with confirmed arthritis exhibited significant localized heat elevation and temperature asymmetry between bilateral measurements. In contrast, healthy joints displayed a more uniform temperature distribution.

## Results

### Ankle analysis

The ankle inflammation cohort comprised *n* = 12 patients, including three bilateral ankle inflammation cases. For the *non-differenced* analysis, each inflamed ankle was treated as an independent sample; consequently, each bilateral case contributed two inflamed ankles, yielding *n* = 15 inflamed ankles. For the *differenced* (contralateral) analysis, only participants with unilateral ankle inflammation were included (*n* = 9), and the three bilateral cases were excluded because contralateral normalization requires an unaffected reference side. In all cases, anterior thermal images were used. The most significant ankle findings are provided in Table 3 of S1 File and are summarized below.

Contralateral differencing (Δf) substantially strengthened the discrimination success for active unilateral ankle inflammation (*n* = 9) by emphasizing within-subject asymmetry. The results show clear thermal asymmetry during active inflammation that is distinguishable from both remission and controls. For example, the difference of maximum temperatures was nearly triple in active inflammation ($1.44^{\circ }$C) than in remission ($0.50^{\circ }$C) and in healthy controls ($0.52^{\circ }$C). However, patients in remission are thermally identical to healthy individuals.

Statistical analysis of raw texture features revealed that skewness is the most significant discriminator for active ankle inflammation. Inflamed ankles exhibited a highly negative skew (−0.889±0.64), which was significantly different from patients in remission (−0.082±0.59,p=0.0014,d=−1.32). The contrast between active inflammation and remission remains the most robust non-differenced texture discriminator in the ankle cohort.

The negative skew suggests that inflammation creates a strong asymmetry with respect to the cooler surrounding tissue. In contrast, clinical remission leads to thermal homogenization, effectively normalizing the skewness to near-zero.

### Wrist analysis

The wrist inflammation cohort comprised *n* = 35 patients with at least one inflamed wrist, including 12 bilateral cases. For the *non-differenced* analysis, each inflamed wrist was treated as an independent sample, yielding *n* = 46 inflamed anterior wrists and *n* = 44 inflamed posterior wrists. For the *differenced* (contralateral) analysis, only participants with unilateral wrist inflammation were included (*n* = 23 anterior, *n* = 21 posterior), and the 12 bilateral cases were excluded because contralateral normalization requires an unaffected reference side. The control group comprised *n* = 38 anterior and *n* = 41 posterior wrists, with remission patients totaling *n* = 18 across all analyses.

#### Latent wrist inflammation.

A subset of 18 wrists from 17 patients underwent MRI within one month of thermal imaging. Clinical examination failed to detect synovitis in any of these joints. However, MRI revealed early inflammatory changes (synovitis, bone marrow edema, or tenosynovitis) in 11 wrists, constituting latent or subclinical inflammation. The remaining seven wrists showed no MRI-detected inflammation and were classified as true negative controls. This MRI-validated cohort enabled evaluation of thermography’s sensitivity to early-stage disease.

In anterior wrists with latent inflammation (see Table 13 in S1 File), raw temperature measurements completely failed to discriminate latent inflammation from healthy controls (all *p* > 0.05). However, texture features achieved large effect sizes. Entropy was most significant (d=−0.83, *p* = 0.007), with inflamed wrists showing reduced entropy compared to controls. Skewness also showed large effect size (*d* = 0.77, *p* = 0.006), and kurtosis achieved *d* = 0.73 (*p* = 0.005). These texture features reveal spatial heterogeneity patterns invisible to absolute temperature measurements, enabling detection of subclinical disease.

Contralateral asymmetry showed minimal discrimination (see Table 6 in S1 File), with only posterior mean temperature difference reaching significance for inflamed versus controls (*d* = 0.870, *p* = 0.020), contrasting sharply with large effects observed in the ankle cohort (*d* > 1.8). This weak lateral asymmetry reflects the bilateral nature of wrist arthritis, even in nominally unilateral cases.

### MCP analysis

Non-differenced analysis revealed no significant discrimination across any temperature or texture metric (all *p* > 0.05). This complete absence of significant findings confirms that raw temperature measurements are insufficient for MCP inflammation detection, and that asymmetry-based features are essential.

Mean temperature L-R asymmetry (see Table 8 in S1 File) emerged as the strongest discriminator for MCP inflammation. In the anterior view, inflamed MCP joints showed $0.63\pm 0.26^{\circ }$C asymmetry compared to $0.29\pm 0.16^{\circ }$C in controls (*p* = 0.0004, *d* = 1.10), representing a large effect size. This asymmetry persisted in clinical remission ($0.57\pm 0.29^{\circ }$C, *p* = 0.014, *d* = 0.90 versus controls), suggesting ongoing subclinical thermal changes. The posterior view confirmed this pattern (*d* = 0.79, *p* = 0.015 for inflamed versus controls). Local entropy L-R asymmetry showed a paradoxical reduction in remission compared to controls (d=−0.64, *p* = 0.047), potentially reflecting thermal homogenization during disease quiescence.

A-P differencing analysis demonstrated the highest discriminative power for MCP inflammation (see Table 9 in S1 File). Skewness A-P asymmetry was the most significant feature, with inflamed MCP joints showing −0.31±0.41 compared to −0.02±0.33 in controls (*p* = 0.0006, d=−0.69). Additional significant A-P features included entropy (*p* = 0.010, *d* = 0.51), kurtosis (*p* = 0.007, d=−0.44), and local entropy (*p* = 0.047, *d* = 0.40), all comparing inflamed versus controls. Notably, mean temperature A-P asymmetry distinguished inflamed from remission (*p* = 0.020, d=−0.64), with remission showing, paradoxically, greater A-P differences than active inflammation.

### Fibromyalgia analysis

#### Thermal characteristics versus healthy controls.

The fibromyalgia cohort comprised *n* = 8 patients. Thermal imaging was performed on both anterior and posterior MCP views. Comparisons were made against healthy controls (*n* = 38) and inflammatory arthritis patients (n=28−41, depending on analysis type). The most significant fibromyalgia findings are summarized in Table 10 of S1 File.

Fibromyalgia patients exhibited significantly cooler posterior MCP temperatures compared to healthy controls, with no detectable thermal asymmetry. The minimum temperature was $27.16\pm 2.48^{\circ }$C in fibromyalgia versus $29.38\pm 2.47^{\circ }$C in controls (*p* = 0.032, d=−0.90), and the maximum temperature was $29.81\pm 2.55^{\circ }$C versus $31.75\pm 2.14^{\circ }$C (*p* = 0.049, d=−0.88), both constituting medium-to-large effect sizes. The anterior MCP view showed no significant thermal differences.

Importantly, fibromyalgia demonstrated a complete absence of thermal asymmetry patterns. L-R differencing revealed no asymmetry in either view across all features (all *p* > 0.05). Similarly, A-P differencing showed no distinctive thermal gradients (all *p* > 0.05). This thermal distribution contrasts sharply with inflammatory arthritis, where mean temperature L-R asymmetry showed large effect sizes (*d* = 1.10 in anterior MCP joints versus controls) and A-P analysis revealed multiple significant features (*d* ranging from 0.40 to 0.69).

#### Fibromyalgia versus inflammatory arthritis.

The comparison between fibromyalgia and inflammatory arthritis patients resulted in significantly discriminative features across all analyses in this study. Raw thermal measurements, particularly in the posterior MCP view, showed large effect sizes that clearly separate these two conditions.

Posterior MCP temperatures showed the most pronounced separation. The maximum temperature was $29.81\pm 2.55^{\circ }$C in fibromyalgia versus $32.44\pm 2.19^{\circ }$C in inflammatory arthritis (*p* = 0.0046, d=−1.17), constituting a large effect size. Similarly, the minimum temperature ($27.16\pm 2.48^{\circ }$C versus $29.90\pm 2.47^{\circ }$C, *p* = 0.007, d=−1.11) and mean temperature ($28.89\pm 2.64^{\circ }$C versus $31.49\pm 2.38^{\circ }$C, *p* = 0.009, d=−1.07) both showed large effect sizes exceeding |*d*| = 1.0.

The anterior view displayed similar patterns with large effect sizes: maximum temperature ($31.16\pm 2.44^{\circ }$C versus $33.13\pm 1.89^{\circ }$C, *p* = 0.020, d=−1.00) and mean temperature ($29.75\pm 3.08^{\circ }$C versus $31.83\pm 2.16^{\circ }$C, *p* = 0.042, d=−0.89). This consistent cooling across all temperature metrics in fibromyalgia patients, approximately 2.5–3 °C below inflammatory arthritis values, provides robust discriminative power.

In contrast to the raw temperature analysis, contralateral differencing showed minimal discrimination between fibromyalgia and inflammatory arthritis. The anterior view revealed no significant differences across all six asymmetry features. The posterior view showed only one marginally significant feature (skewness difference, *p* = 0.019, d=−0.53), with a medium effect size far smaller than the temperature differences observed in the raw analysis.

### Knee analysis

Thermal imaging was performed on the anterior knee view only; posterior view data were not collected. The cohort included four clinical groups: healthy controls (*n* = 58), active knee inflammation (*n* = 21), clinical remission (*n* = 17), and OA (*n* = 5).

Non-differenced analysis revealed no significant discrimination across any temperature or texture metric (all *p* > 0.05). On the other hand, differenced analysis showed strong discrimination (see Table 11 in S1 File). Mean temperature asymmetry showed a very large effect size of *d* = 2.15 (*p* < 0.001), with inflamed knees exhibiting $1.16\pm 0.66^{\circ }$C asymmetry compared to $0.29\pm 0.26^{\circ }$C in controls. Additional significant differenced features are: mean temperature difference (*d* = 2.15, *p* < 0.001), maximum temperature difference (*d* = 1.28, *p* < 0.001), entropy difference (*d* = 1.00, *p* = 0.003), local entropy difference (*d* = 0.66, *p* = 0.019), and skewness difference (*d* = 0.78, *p* = 0.027). The consistency across multiple features and the large effect sizes indicate that knee inflammation produces marked and robust thermal asymmetry that can be reliably detected.

Clinical remission showed significantly reduced thermal asymmetry compared to active inflammation. Mean temperature asymmetry in remission ($0.47\pm 0.50^{\circ }$C) was significantly lower than active inflammation ($1.16\pm 0.66^{\circ }$C, *p* < 0.001, *d* = 1.16), although still slightly elevated compared to controls. Only one feature remained significantly different between remission and controls: local entropy difference (*p* = 0.022, *d* = 0.52).

#### Osteoarthritis (OA).

OA patients (*n* = 5) showed minimal thermal asymmetry (see Table 12 in S1 File), indistinguishable from healthy controls (all *p* > 0.05). However, OA demonstrated significantly lower asymmetry than inflammatory arthritis across three features: mean temperature difference (d=−1.49, *p* = 0.001), entropy difference (d=−0.98, *p* = 0.028), and maximum temperature difference (d=−1.07, *p* = 0.034).

This pattern suggests that thermal asymmetry could differentiate inflammatory from degenerative arthritis. Inflammatory arthritis produces marked asymmetry through focal synovitis and hyperemia, while the degenerative disease remains symmetric. The small OA sample (*n* = 5) limits definitive conclusions and necessitates validation in larger cohorts, but the large effect sizes and consistent pattern across multiple features support this interpretation.

### Latent inflammation

#### MCP joints.

MCP analysis included anterior and posterior images from the latent (sub-clinical) inflammation group (*n* = 11) and healthy controls (*n* = 38). Non-differenced analysis revealed no significant discrimination across any temperature or texture metric (all *p* > 0.05) for both anterior and posterior images.

While absolute temperature and texture parameters from individual anterior or posterior views showed no significant differences (*p* > 0.05), A-P differencing analysis revealed that inflammation significantly affects the A-P entropy difference. The mean entropy A-P difference was 0.449±0.318 in the inflammation group versus 0.099±0.591 in healthy controls (*p* = 0.045, *d* = 0.641, medium effect). This finding suggests that inflammatory arthritis creates distinctive spatial thermal patterns in MCP joints that are detectable through A-P analysis.

#### Wrist.

Wrist analysis included anterior and posterior images of the inflammation group (*n* = 18) and healthy controls (*n* = 38). Both absolute measurements and texture features were analyzed. In anterior wrists, texture parameters were the most significant (see Table 13 in S1 File). No significant parameter was found in posterior wrists.

A-P differencing analysis revealed highly significant differences between inflammation and healthy controls. Healthy controls exhibited significantly larger entropy differences (1.074±0.616) compared to inflammation patients (0.359±0.709,p=0.001,d=−1.105). This paradoxical finding suggests that inflammation homogenizes local thermal patterns, reducing spatial differences despite increasing absolute thermal heterogeneity.

## Discussion

This study provides evidence that consumer-grade thermal cameras, augmented with texture-based feature extraction and A-P differencing, can detect joint inflammation in arthritis (see Table 14 in S1 File). These two augmentations are the core contributions of this work and aim to address limitations in existing thermographic approaches.

While conventional thermography relies on absolute temperature or simple summary statistics, our results show that spatial texture descriptors, particularly entropy, skewness, and kurtosis, provide complementary and often superior diagnostic information. The clearest evidence comes from latent wrist inflammation, where absolute temperature measurements completely failed to discriminate inflamed from healthy joints (all *p* > 0.05), yet texture features achieved large effect sizes. Anterior wrist entropy showed d=−0.83 (*p* = 0.007), skewness reached *d* = 0.77 (*p* = 0.006), and kurtosis achieved *d* = 0.73 (*p* = 0.005). The physiological basis for this discriminative power lies in the spatial heterogeneity of inflammatory heat distribution. Active synovitis does not produce uniform warming but creates focal hot spots interspersed with cooler regions, reflecting heterogeneous synovial proliferation and variable hyperemia. Entropy captures this overall thermal heterogeneity, while skewness detects asymmetric patterns. For example, inflamed ankles showed strongly negative skew (−0.89±0.64) compared to remission (−0.08±0.59; d=−1.32, *p* = 0.001), indicating intense hot spots embedded in predominantly cooler backgrounds. Kurtosis identifies peaked distributions, with elevated values in wrist remission (*d* = 0.80, *p* = 0.012) suggesting residual focal warming despite clinical improvement. Texture features also revealed residual thermal abnormalities in clinical remission, masked by normalized absolute temperatures. Wrist remission showed reduced local entropy (d=−0.73, *p* = 0.001) yet elevated kurtosis (*d* = 0.80) and skewness (*d* = 0.81), a signature distinct from both active inflammation and healthy controls, suggesting thermal remodeling during disease quiescence or subclinical synovitis.

Texture features alone, however, cannot distinguish bilateral disease from bilateral warmth in healthy joints, because conventional contralateral comparison requires one unaffected limb. Building on these findings, A-P differencing is a novel normalization strategy that extends thermographic utility to bilateral presentations. This is especially relevant for rheumatoid arthritis and other symmetric inflammatory conditions. The wrist analysis illustrates this: contralateral differencing achieved minimal discrimination (only posterior mean temperature reached significance, *d* = 0.87, *p* = 0.020), reflecting bilateral wrist arthritis. In contrast, A-P differencing yielded large effect sizes for detecting latent inflammation, with an A-P entropy difference of d=−1.11 (*p* = 0.001). The direction of this finding, that inflammation reduces rather than increases A-P asymmetry, motivates a working hypothesis. One plausible account is that healthy wrists maintain substantial A-P thermal gradients because of anatomical asymmetry: the dorsal surface lies close to the bone and synovium with minimal soft tissue buffering, whereas the palmar surface contains deeper flexor tendons and neurovascular bundles. Under this hypothesis, inflammation could attenuate the gradient by warming both surfaces toward equilibrium through diffuse hyperemia and concurrent tenosynovitis. This mechanism is speculative and was not directly probed by our imaging protocol; dedicated measurements (e.g., paired ultrasound/MRI of dorsal and palmar compartments) are needed to test it. Notably, A-P patterns showed joint-specific directionality. MCP joints with latent inflammation exhibited a pattern opposite to the wrist: an increased A-P entropy difference (0.45±0.32 versus 0.10±0.59 in controls; *d* = 0.64, *p* = 0.045). One explanation, as yet unvalidated, invokes anatomical differences: MCP joints have discrete, dorsally-prominent synovial recesses with less palmar tissue depth, so early inflammation might preferentially warm the dorsal surface while palmar temperatures remain buffered, amplifying rather than reducing A-P asymmetry. This joint-specific directionality, like the wrist hypothesis, requires validation through anatomically-localized imaging. The combination of A-P differencing and texture features was especially effective. For latent wrist inflammation, A-P local entropy achieved d=−1.10 (*p* = 0.004), A-P kurtosis reached *d* = 0.52 (*p* = 0.012), and A-P skewness yielded *d* = 0.65 (*p* = 0.016), all capturing subtle inflammatory signatures invisible to single-view temperature measurements.

Together, texture features and A-P differencing span the full spectrum of arthritis presentations: unilateral disease through contralateral differencing with texture features (knee *d* = 2.15, ankle *d* = 1.83), bilateral disease through A-P differencing with texture features (wrist d=−1.11), and early or subtle disease through texture-only analysis. A-P imaging adds only a modest acquisition overhead. These findings suggest that texture-enhanced thermography with dual differencing strategies may complement conventional arthritis assessment as an exploratory tool, though validation in independent cohorts is needed before clinical translation. Several limitations warrant consideration. Manual ROI segmentation remains operator-dependent, and automated segmentation would enhance reproducibility. The A-P analysis was limited to joints with both views acquired (wrists and MCP joints); future studies should implement multi-view protocols across all joint sites. The opposing directionality of A-P patterns between wrists and MCP joints requires validation in independent cohorts and mechanistic investigation through anatomically-localized MRI. Longitudinal studies are needed to assess whether texture-enhanced A-P thermography can track disease progression and treatment response.

Finally, our findings extend recent literature on thermographic assessment of rheumatic diseases. The systematic review by Nallathambi et al. reported that IRT is effective for diagnosing active synovitis across multiple conditions [15]. Our effect sizes for knee (*d* = 2.15) and ankle (*d* = 1.83) inflammation are consistent with these performance estimates. To our knowledge, the use of A-P thermal differencing for arthritis detection has not been previously reported. While prior studies have analyzed dorsal and palmar hand surfaces separately [6], the explicit comparison of A-P thermal gradients as a derived feature represents a novel contribution. The large effect size for A-P entropy difference in latent wrist inflammation (d=−1.11) exceeds that of any single-view absolute measurement in this cohort, suggesting that this approach warrants further investigation. The joint-specific directionality of A-P patterns (opposite signs for wrist versus MCP) also implies that normative A-P reference values may need to be established separately for each anatomical site.

## Conclusions

This study demonstrates that texture-based feature extraction and A-P differencing greatly expand the diagnostic capability of consumer-grade thermal imaging for arthritis. Texture features capture inflammatory heterogeneity invisible to absolute temperature measurements, enabling detection of early and subclinical disease. A-P differencing extends the applicability to bilateral presentations where contralateral comparison fails, with joint-specific patterns reflecting underlying anatomical differences. These methodological innovations, combined with the accessibility and low cost of consumer-grade cameras, position texture-enhanced thermography with dual differencing as a viable screening tool to supplement clinical evaluation, aid treatment monitoring, and support differential diagnosis.

## Supporting information

S1 File Supplementary tables. Detailed statistical results supporting all analyses in the main text. The file contains, in order: the clinical-diagnosis distribution of the cohort and the accrued MRI evaluations; ankle findings; wrist analyses (absolute, contralateral L-R, and anterior-posterior A-P features for anterior and posterior views); MCP joint analyses (L-R and A-P differencing); fibromyalgia comparisons; knee analyses (contralateral differencing and the osteoarthritis comparison); latent (subclinical) wrist inflammation; and a summary of the most significant findings. Individual tables are numbered as they appear in the file and are cited by number in the main text.
(PDF)
