# Supplementary material for: Texture-enhanced thermography for joint inflammation detection using consumer-grade thermal cameras
Source: PLoS One. 2026 Jul 29;21(7):e0354709. doi: 10.1371/journal.pone.0354709 (PMC13419199; doi:10.1371/journal.pone.0354709)
Supplement: S1 File — Detailed statistical results supporting all analyses in the main text. The file contains, in order: the clinical-diagnosis distribution of the cohort and the accrued MRI evaluations; ankle findings; wrist analyses (absolute, contralateral L-R, and anterior-posterior A-P features for anterior and posterior views); MCP joint analyses (L-R and A-P differencing); fibromyalgia comparisons; knee analyses (contralateral differencing and the osteoarthritis comparison); latent (subclinical) wrist inflammation; and a summary of the most significant findings. Individual tables are numbered as they appear in the file and are cited by number in the main text. (PDF) [file pone.0354709.s001.pdf]

Supplementary Material

Abstract

This file provides supplementary tables. Table values are sorted by significance (p-values).

Contents

1 Study Cohort **3**

2 Ankle Analysis **4**

3 Wrist Analysis **5**

4 MCP Joints **9**

5 Fibromyalgia Analysis - MCP **11**

6 Knee Analysis **12**

7 Latent Inflammation - Wrists **14**

8 Summary **15**

List of Tables

1 Clinical diagnoses . . . 3
2 Accured MRI evaluations. . . . 3
3 Ankle analysis . . . 4
4 Wrist analysis - absolute features (anterior) . . . 5
5 Wrist analysis - absolute features (posterior) . . . 6
6 Wrist analysis - L-R asymmetry . . . 7
7 Wrist analysis - A-P asymmetry (absolute features) . . . 8
8 MCP analysis - L-R asymmetry . . . 9
9 MCP analysis - A-P asymmetry . . . 10
10 Fibromyalgia analysis - MCP . . . 11
11 Knee joints - L-R asymmetry . . . 12
12 Knee joints - knee inflammation and OA . . . 13

13 Latent inflammation - wrists 14
14 Summary of the findings 15

1 Study Cohort

Table 1: Frequency and percentage distribution of clinical diagnoses in the study cohort ($N = 239$).

**Diagnosis**	**N**	**Percentage**
Healthy Control	72	30.1%
Rheumatoid Arthritis (RA)	68	28.5%
Psoriatic Arthritis (PSA)	25	10.5%
Fibromyalgia	23	9.6%
Osteoarthritis (OA)	20	8.4%
Gout	13	5.4%
Inflammatory OA (IOA)	5	2.1%
Other/Unspecified	5	2.1%
CPPD/Pseudogout	4	1.7%
Inflammatory (Unspecified)	3	1.3%
Familial Mediterranean Fever (FMF)	1	0.4%
**Total**	**239**	**100.0%**

Table 2: Accured MRI evaluations.

**Area of hand analyzed**	**Number of patients**	**Note**
Wrist	17	One bilateral patient
MCPs	11	
Knee	2	Not used due to a small sample size
Ankle	1	Not used due to a small sample size

2 Ankle Analysis

Table 3: Key ankle findings. Contralateral differencing highlights temperature asymmetry and entropy as the strongest discriminators of active unilateral ankle inflammation, while clinical remission is indistinguishable from that of healthy controls. Non-differenced texture analysis identifies skewness as the main discriminator between inflammation and remission.

**Metric**	**Group A**	**Group B**	p**-value**	d	**Sig**
**Differenced: Unilateral inflammation (n=9), Remission (n=19), Controls (n=58)**					
Inflamed vs. Control					
Max temp difference (°C)	1.44 ± 0.93	0.52 ± 0.41	0.0005	1.83	***
Mean temp difference (°C)	1.30 ± 1.05	0.44 ± 0.33	0.0053	1.80	**
Entropy difference	0.63 ± 0.50	0.27 ± 0.20	0.0087	1.42	**
Inflamed vs. Remission					
Max temp difference (°C)	1.44 ± 0.93	0.50 ± 0.40	0.0019	1.53	**
Entropy difference	0.63 ± 0.50	0.22 ± 0.20	0.0079	1.26	**
Mean temp difference (°C)	1.30 ± 1.05	0.51 ± 0.44	0.0388	1.15	*
Remission vs. Controls: no significant differences for any metric (all $p > 0.18$, $\|d\| < 0.25$).					
**Non-differenced: Inflamed ankles (n=15), Remission (n=19), Controls (n=58)**					
Inflamed vs. Controls					
Skewness	-0.889 ± 0.637	-0.564 ± 0.812	0.0635	-0.42	NS
Inflamed vs. Remission					
Skewness	-0.889 ± 0.637	-0.082 ± 0.588	0.0014	-1.32	**
Remission vs. Controls					
Skewness	-0.082 ± 0.588	-0.564 ± 0.812	0.0322	0.63	*
Energy	0.021 ± 0.013	0.015 ± 0.011	0.0286	0.57	*

Values are mean ± SD.
Significance: *** $p < 0.001$, ** $p < 0.01$, * $p < 0.05$, NS stands for non-significant.
Effect sizes: small ($0.2 \leq |d| < 0.5$), medium ($0.5 \leq |d| < 0.8$), large ($0.8 \leq |d| < 1.2$), very large ($1.2 \leq |d|$).

3 Wrist Analysis

Table 4: Statistical comparison of absolute thermal and texture features for anterior wrist. All cases included (bilateral cases treated as independent samples). Remission demonstrates a distinct texture signature with reduced local entropy but elevated kurtosis and skewness compared to controls.

**Metric**	**Group A**	**Group B**	**p-value**	d	**Sig**
**Anterior Wrist: Inflamed (n=46), Remission (n=18), Controls (n=38)**					
Remission vs. Control					
Local Entropy	2.552 ± 0.204	2.715 ± 0.251	0.0009	-0.726	***
Kurtosis	0.027 ± 0.043	0.015 ± 0.012	0.0118	0.798	*
Skewness	0.188 ± 0.157	0.135 ± 0.068	0.0174	0.813	*
Entropy	5.795 ± 0.453	6.158 ± 0.522	0.0316	-0.672	*
Inflamed vs. Remission					
Local Entropy	2.643 ± 0.227	2.552 ± 0.204	0.0321	0.411	*
Max temp (°C)	34.066±2.123	33.669±1.769	0.0415	0.281	*
Inflamed vs. Control					
Local Entropy	2.643 ± 0.227	2.715 ± 0.251	0.0367	-0.310	*

Values are mean ± SD.
Bilateral cases included; each wrist treated as independent sample.
Significance: *** $p < 0.001$, ** $p < 0.01$, * $p < 0.05$.
Effect sizes: small ($0.2 \leq |d| < 0.5$), medium ($0.5 \leq |d| < 0.8$), large ($0.8 \leq |d| < 1.2$), very large ($1.2 \leq |d|$).

Table 5: Statistical comparison of absolute thermal and texture features for posterior wrist. All cases included (bilateral cases treated as independent samples). Inflamed wrists show elevated temperatures across all metrics (mean, max, min) compared to both controls and remission.

Metric	Group A	Group B	p-value	d	Sig
**Posterior Wrist: Inflamed (n=44), Remission (n=18), Controls (n=41)**					
Inflamed vs. Remission					
Mean temp (°C)	32.729±2.061	31.243±2.330	0.0047	0.565	**
Max temp (°C)	33.492±2.008	32.370±2.195	0.0059	0.497	**
Min temp (°C)	31.070±2.335	29.640±2.651	0.0204	0.464	*
Inflamed vs. Control					
Max temp (°C)	33.492±2.008	32.661±2.159	0.0063	0.489	**
Mean temp (°C)	32.729±2.061	31.695±2.282	0.0082	0.497	**
Remission vs. Control					
Entropy	5.394 ± 0.468	5.036 ± 0.505	0.0292	0.680	*
Kurtosis	0.036 ± 0.047	0.064 ± 0.073	0.0317	-0.422	*
Skewness	0.228 ± 0.246	0.371 ± 0.370	0.0437	-0.462	*

Values are mean ± SD.
Bilateral cases included; each wrist treated as independent sample.
Significance: *** $p < 0.001$, ** $p < 0.01$, * $p < 0.05$.
Effect sizes: small ($0.2 \le |d| < 0.5$), medium ($0.5 \le |d| < 0.8$), large ($0.8 \le |d| < 1.2$), very large ($1.2 \le |d|$).

Table 6: Statistical comparison of differenced (contralateral) thermal and texture features for unilateral wrist inflammation. Bilateral cases were excluded to isolate true lateral asymmetry. Minimal asymmetry observed in the anterior view; the posterior view shows significant mean temperature asymmetry for inflamed wrists with a large effect size.

**Metric**	**Group A**	**Group B**	***p*-value**	*d*	**Sig**
**Anterior Wrist: Inflamed (n=23), Remission (n=18), Controls (n=38)**					
No significant differences for any comparison (all $p > 0.05$*).*					
**Posterior Wrist: Inflamed (n=21), Remission (n=18), Controls (n=41)**					
Inflamed vs. Control					
Mean temp diff (°C)	0.820 ± 0.846	0.354 ± 0.335	0.0195	0.870	*
Remission vs. Control					
Skewness diff	0.054 ± 0.178	0.174 ± 0.254	0.0473	-<br>0.579	*
Inflamed vs. Remission: no significant differences (all $p > 0.05$*).*					

Values are mean ± SD.
Bilateral wrist cases excluded from this analysis (n=12 excluded).
Significance: *** $p < 0.001$, ** $p < 0.01$, * $p < 0.05$.
Effect sizes: small ($0.2 \leq |d| < 0.5$), medium ($0.5 \leq |d| < 0.8$), large ($0.8 \leq |d| < 1.2$), very large ($1.2 \leq |d|$).

Table 7: Statistical comparison of A-P asymmetry for absolute wrist features. All paired cases included (bilateral treated as independent). A-P differencing reveals highly significant patterns with very large effect sizes, particularly for entropy and mean temperature differences, distinguishing all three groups.

**Metric**	**Group A**	**Group B**	**p-value**	d	**Sig**
**A-P Asymmetry (Absolute): Inflamed (n=44), Remission (n=18), Controls (n=38)**					
Inflamed vs. Remission					
A-P Mean temp (°C)	0.203 ± 0.955	0.876 ± 0.856	0.0001	-1.067	***
A-P Entropy	0.748 ± 0.677	0.281 ± 0.629	0.0042	0.821	**
A-P Skewness	-0.142 ± 0.298	-0.019 ± 0.164	0.0028	-0.718	**
A-P Kurtosis	-0.028 ± 0.061	-0.004 ± 0.050	0.0049	-0.550	**
A-P Min temp (°C)	-0.214 ± 1.235	0.481 ± 1.223	0.0090	-0.711	**
Inflamed vs. Control					
A-P Mean temp (°C)	0.203 ± 0.955	0.831 ± 0.863	0.0003	-0.760	***
A-P Local Entropy	0.203 ± 0.384	0.393 ± 0.303	0.0004	-0.785	***
A-P Max temp (°C)	0.559 ± 1.020	0.977 ± 0.959	0.0279	-0.451	*
A-P Min temp (°C)	-0.214 ± 1.235	0.354 ± 1.040	0.0352	-0.414	*
Remission vs. Control					
A-P Entropy	0.281 ± 0.629	1.144 ± 0.635	0.0005	-1.164	***
A-P Kurtosis	-0.004 ± 0.050	-0.051 ± 0.068	0.0006	0.627	***
A-P Skewness	-0.019 ± 0.164	-0.213 ± 0.278	0.0006	0.798	***
A-P Local Entropy	0.261 ± 0.293	0.393 ± 0.303	0.0037	-1.076	**

Values are mean ± SD. A-P = Anterior minus Posterior.
Bilateral cases included; paired samples with both views.
Significance: *** $p < 0.001$, ** $p < 0.01$, * $p < 0.05$.
Effect sizes: small ($0.2 \leq |d| < 0.5$), medium ($0.5 \leq |d| < 0.8$), large ($0.8 \leq |d| < 1.2$), very large ($1.2 \leq |d|$).

4 MCP Joints

Table 8: Statistical comparison of contralateral (L-R) differenced thermal and texture features for unilateral MCP inflammation. Bilateral cases excluded. Mean temperature asymmetry emerged as the strongest discriminator with a large effect size.

**Metric**	**Group A**	**Group B**	***p*-value**	*d*	**Sig**
**Anterior MCP: Inflamed (n=28), Remission (n=18), Controls (n=38)**					
Inflamed vs. Control					
Mean temp difference (°C)	0.63 ± 0.26	0.29 ± 0.16	0.0004	1.10	***
Remission vs. Control					
Mean temp difference (°C)	0.57 ± 0.29	0.29 ± 0.16	0.0137	0.90	*
Local entropy difference	0.05 ± 0.04	0.08 ± 0.05	0.0465	-0.64	*
Inflamed vs. Remission: no significant differences (all $p > 0.05$).					
**Posterior MCP: Inflamed (n=28), Remission (n=17), Controls (n=38)**					
Inflamed vs. Control					
Mean temp difference (°C)	0.65 ± 0.38	0.32 ± 0.21	0.0145	0.79	*
Other comparisons: no significant differences (all $p > 0.05$).					

Values are mean ± SD.
Bilateral MCP cases excluded from this analysis (unilateral only, n=84-83).
Significance: *** $p < 0.001$, ** $p < 0.01$, * $p < 0.05$.
Effect sizes: small ($0.2 \leq |d| < 0.5$), medium ($0.5 \leq |d| < 0.8$), large ($0.8 \leq |d| < 1.2$), very large ($1.2 \leq |d|$).
m1-m2 (mean temperature L-R asymmetry) is the most robust discriminator across views.

Table 9: Statistical comparison of A-P differenced thermal and texture features for MCP joints. Paired analysis of patients with both views. A-P texture asymmetry showed the highest discrimination, with multiple significant features distinguishing inflammation from controls.

**Metric**	**Group A**	**Group B**	**p-value**	d	**Sig**
**A-P Asymmetry: Inflamed (n=66), Remission (n=17), Controls (n=38)**					
Inflamed vs. Control					
Skewness A-P diff	-0.31 ± 0.41	-0.02 ± 0.33	0.0006	-0.69	***
Entropy A-P diff	0.42 ± 0.66	0.10 ± 0.52	0.0100	0.51	*
Kurtosis A-P diff	-0.15 ± 0.37	0.01 ± 0.27	0.0070	-0.44	**
Local entropy A-P diff	0.19 ± 0.21	0.10 ± 0.17	0.0468	0.40	*
Inflamed vs. Remission					
Mean temp A-P diff (°C)	0.29 ± 0.90	0.85 ± 0.85	0.0198	-0.64	*
Remission vs. Control: no significant differences (all $p > 0.05$).					

Values are mean ± SD. A-P diff = Anterior value - Posterior value.
Bilateral cases included; paired samples with both hands.
Significance: *** $p < 0.001$, ** $p < 0.01$, * $p < 0.05$.
Effect sizes: small ($0.2 \leq |d| < 0.5$), medium ($0.5 \leq |d| < 0.8$), large ($0.8 \leq |d| < 1.2$), very large ($1.2 \leq |d|$).

5 Fibromyalgia Analysis - MCP

Table 10: Statistical comparison of fibromyalgia patients with healthy controls and inflammatory arthritis patients in MCP joints. Fibromyalgia exhibits significantly cooler posterior temperatures with large effect sizes, representing strong discrimination. The absence of thermal asymmetry distinguishes fibromyalgia from inflammatory conditions.

**Metric**	**Group A**	**Group B**	**p-value**	d	**Sig**
**Fibromyalgia (n=8) vs. Control (n=38) Non-differenced**					
Posterior MCP					
Min Temperature (°C)	27.16 ± 2.48	29.38 ± 2.47	0.0320	-0.90	*
Max Temperature (°C)	29.81 ± 2.55	31.75 ± 2.14	0.0491	-0.88	*
Anterior MCP: no significant differences (all $p > 0.05$).					
Differenced analysis (both views): no significant differences (all $p > 0.05$).					
**Fibromyalgia (n=8) vs. Inflammatory Arthritis: Non-differenced**					
Anterior MCP (Fibro n=8, Inflamed n=41)					
Max Temperature (°C)	31.16 ± 2.44	33.13 ± 1.89	0.0201	-1.00	*
Mean Temperature (°C)	29.75 ± 3.08	31.83 ± 2.16	0.0423	-0.89	*
Posterior MCP (Fibro n=8, Inflamed n=40)					
Max Temperature (°C)	29.81 ± 2.55	32.44 ± 2.19	0.0046	-1.17	**
Min Temperature (°C)	27.16 ± 2.48	29.90 ± 2.47	0.0070	-1.11	**
Mean Temperature (°C)	28.89 ± 2.64	31.49 ± 2.38	0.0085	-1.07	**
**Fibromyalgia (n=8) vs. Inflammatory Arthritis (n=28): Differenced**					
Posterior MCP					
Skewness difference	0.119 ± 0.212	0.259 ± 0.276	0.0191	-0.53	*

Values are mean ± SD.
Negative Cohen's d indicates fibromyalgia patients have lower values.
Posterior MCP temperatures vs. inflammatory arthritis show large effect sizes ($|d| > 1.0$).
Significance: *** $p < 0.001$, ** $p < 0.01$, * $p < 0.05$.
Effect sizes: small ($0.2 \leq |d| < 0.5$), medium ($0.5 \leq |d| < 0.8$), large ($0.8 \leq |d| < 1.2$), very large ($1.2 \leq |d|$).

6 Knee Analysis

Table 11: Statistical comparison of differenced (contralateral) thermal features for knee joints. Mean temperature asymmetry strongly discriminates between active inflammation and healthy controls, with a very large effect size observed in this study ($d = 2.15$), demonstrating exceptional sensitivity. Temperature asymmetry also effectively distinguishes active inflammation from remission.

**Metric**	**Group A**	**Group B**	p**-value**	d	**Sig**
**Anterior Knee: Inflammation (n=21), Remission (n=17), Controls (n=58)**					
Inflammation vs. Control					
Mean temp difference	1.160 ± 0.664	0.292 ± 0.257	0.0000	2.15	***
Max temp difference	0.838 ± 0.528	0.386 ± 0.265	0.0003	1.28	***
Entropy difference	0.471 ± 0.385	0.234 ± 0.157	0.0030	1.00	**
Local Entropy difference	0.089 ± 0.070	0.052 ± 0.050	0.0189	0.66	*
Skewness difference	0.044 ± 0.034	0.024 ± 0.020	0.0268	0.78	*
Remission vs. Control					
Local Entropy difference	0.078 ± 0.053	0.052 ± 0.050	0.0224	0.52	*
Inflammation vs. Remission					
Mean temp difference	1.160 ± 0.664	0.466 ± 0.501	0.0007	1.16	***
Max temp difference	0.838 ± 0.528	0.493 ± 0.430	0.0428	0.71	*

Values are mean ± SD for differenced features (L-R asymmetry).
Mean temperature difference in inflammation vs. control shows $d = 2.15$, very large effect size.
Significance: *** $p < 0.001$, ** $p < 0.01$, * $p < 0.05$.
Effect sizes: small ($0.2 \leq |d| < 0.5$), medium ($0.5 \leq |d| < 0.8$), large ($0.8 \leq |d| < 1.2$), very large ($1.2 \leq |d|$).

Table 12: Inflammatory arthritis shows significantly greater asymmetry than OA, enabling differential diagnosis.

Metric	Group A	Group B	p-value	d	Sig
**OA (n=5) vs. Inflammation (n=21): Differenced**					
Mean temp difference	0.248 ± 0.220	1.160 ± 0.664	0.0013	-1.49	**
Entropy difference	0.126 ± 0.084	0.471 ± 0.385	0.0284	-0.98	*
Max temp difference	0.309 ± 0.255	0.838 ± 0.528	0.0342	-1.07	*
Clinical interpretation: Inflammatory arthritis shows much greater thermal asymmetry than osteoarthritis, enabling differential diagnosis. Small OA sample limits power.					
**OA (n=5) vs. Control (n=58): Differenced**					
No significant differences (all $p > 0.05$). OA shows symmetric thermal pattern.					

Values are mean ± SD for differenced features (L-R asymmetry).
Negative Cohen's d indicates Group A has lower asymmetry than Group B.
OA sample size (n=5) limits statistical power; findings are exploratory.
Significance: *** $p < 0.001$, ** $p < 0.01$, * $p < 0.05$.
Effect sizes: small ($0.2 \leq |d| < 0.5$), medium ($0.5 \leq |d| < 0.8$), large ($0.8 \leq |d| < 1.2$), very large ($1.2 \leq |d|$).

7 Latent Inflammation - Wrists

Table 13: Statistical comparison of texture parameters between latent wrist inflammation ($n = 18$) and control groups ($n = 38$). Texture features demonstrate significant differences, whereas absolute temperature metrics remain indistinguishable.

**Parameter**	**Inflammation**	**Control**	***p*-value**	**Cohen's *d***	**Sig.**
Anterior (non-differenced)					
Entropy	5.711 ± 0.541	6.171 ± 0.563	0.007	-0.828	**
Local Entropy	2.546 ± 0.218	2.710 ± 0.247	0.011	-0.689	*
Kurtosis	0.041 ± 0.037	0.021 ± 0.021	0.005	0.727	**
Skewness	0.238 ± 0.135	0.158 ± 0.086	0.006	0.769	**
A-P Differenced					
Entropy	0.359 ± 0.709	1.074 ± 0.616	0.001	-1.105	**
Local Entropy	0.162 ± 0.275	0.400 ± 0.184	0.004	-1.098	**
Kurtosis	-0.030 ± 0.086	-0.110 ± 0.175	0.012	0.524	*
Skewness	-0.093 ± 0.253	-0.285 ± 0.316	0.016	0.645	*

Values are mean ± SD.
Significance: *** $p < 0.001$, ** $p < 0.01$, * $p < 0.05$.
Effect sizes: small ($0.2 \leq |d| < 0.5$), medium ($0.5 \leq |d| < 0.8$), large ($0.8 \leq |d| < 1.2$), very large ($1.2 \leq |d|$).

8 Summary

Table 14: Summary of the most significant findings

Joint	Analysis Type	Key Finding	Effect Size (d)	p-value	Clinical Significance
**Knee**	Contralateral difference	Mean temp asymmetry: 1.16°C (inflamed) vs. 0.29°C (control)	2.15	<0.001	Strongest discriminator in study
**Ankle**	Contralateral difference	Max temp asymmetry: 1.44°C (inflamed) vs. 0.52°C (control)	1.83	0.0005	Very large effect for unilateral inflammation
**Ankle**	Non-differenced	Skewness: −0.89 (inflamed) vs. −0.08 (remission)	−1.32	0.001	Distinguishes active from remission
**Wrist**	A-P difference	Entropy diff: 0.36 (latent inflam.) vs. 1.07 (control)	−1.11	0.001	Detects subclinical inflammation
**MCP**	Contralateral difference	Mean temp asymmetry: 0.63°C (inflamed) vs. 0.29°C (control)	1.10	0.0004	Large effect for small joint
**Knee**	Inflammatory vs. OA	Mean temp difference	−1.49	0.001	Differentiates inflammatory from degenerative
**MCP**	Inflammatory vs. Fibromyalgia	Max temp: 32.44°C (IA) vs. 29.81°C (fibro)	−1.17	0.005	Fibromyalgia shows cooling vs. warming

Effect sizes: small ($0.2 \leq |d| < 0.5$), medium ($0.5 \leq |d| < 0.8$), large ($0.8 \leq |d| < 1.2$), very large ($1.2 \leq |d|$)
